# Comparison of proteins with anti-influenza virus effects in parotid and submandibular-sublingual saliva in humans

**DOI:** 10.1186/s12903-022-02686-1

**Published:** 2022-12-24

**Authors:** Kenkichi Yamamoto, Shinji Yamamoto

**Affiliations:** Personal Health Care Products Research Laboratories, Kao Corporation, 2-1-3 Bunka, Sumida-ku, 131-8501 Tokyo, Japan

**Keywords:** Antiviral agents, Influenza A virus, N-Acetylneuraminic acid, Mucins, Cystatins

## Abstract

**Background:**

Saliva possesses antiviral activity, with submandibular-sublingual (SMSL) saliva having higher antiviral activity than parotid saliva. Various salivary proteins have inactivating effects on influenza A virus (IAV), but the detailed relationship between antiviral proteins and salivary anti-IAV activities in the parotid and SMSL glands is unknown. Here, to identify salivary proteins with anti-IAV activity, salivary proteins from parotid and SMSL glands were identified, quantified, and compared using liquid chromatography-mass spectrometry.

**Methods:**

Twelve healthy male volunteers participated in the study. Parotid and SMSL saliva was collected by suction and collection devices. We assessed anti-IAV activities, protein concentrations, and protein-bound sialic acid concentrations in parotid and SMSL saliva.

**Results:**

SMSL had significantly higher anti-IAV activity than parotid saliva. SMSL also had higher concentrations of glycoproteins, such as mucin 5B and mucin 7, protein-bound sialic acid, cystatins, and lysozyme C, compared with parotid saliva. Salivary mucin 5B and mucin 7 concentrations significantly positively correlated with the salivary protein-bound sialic acid concentration. Salivary anti-IAV activity significantly positively correlated with protein-bound sialic acid, mucin 5B, mucin 7, cystatin-C, -S, and -SN concentrations.

**Conclusion:**

Salivary mucins, cystatins, and lysozyme C contribute to the high anti-IAV activity of SMSL saliva.

## Introduction

Humans have 3 main paired salivary glands, the parotid, submandibular, and sublingual glands, which differ in their secretion type: serous, seromucous, and mucous, respectively. Multiple minor glands are also present underneath the oral mucosa. Saliva is considered important for overall health as well as oral health, and conditions in which salivary secretion is reduced are associated with oral disease and discomfort [1, 2]. Proteins are abundant in saliva and contribute to oral integrity, digestion, lubrication, and buffering, as well as antimicrobial activity. These proteins include mucins, cystatins, histatins, statherins, amylases, and proline-rich proteins, with the protein composition varying among the gland types [3–5].

Influenza is a serious infectious viral disease caused by viruses A, B, C, and D that affects a large number of people globally. Influenza A and B viruses cause seasonal disease epidemics almost every year. The World Health Organization estimates that 300–500 million infections and 290,000–650,000 deaths per year are due to influenza [6, 7]. Of the A and B viruses, influenza A virus (IAV) warrants special attention as it infects a very wide range of hosts and has caused several pandemics, including the Spanish flu in 1918 [8].

Hyposalivation is associated with acute respiratory infection [9] including diseases such as the influenza and recently COVID-19 [10, 11]. Salivary proteins, including cystatins, lactoferrin, lysozyme, mucins, and scavenger receptor cysteine-rich glycoprotein-340 (gp-340), exhibit both antimicrobial and antiviral activities [12–14]. Various factors in saliva (e.g., sialic acid-containing mucin 5B, gp-340, and histatin-1), are reported to have potent inhibitory activities against IAV [15]. Kobayashi et al. reported that the anti-IAV activity of saliva broadly varies among individuals and demonstrated that anti-IAV activity positively correlates with the concentration of protein-bound sialic acid [16]. Findings reported by Limsuwat et al. indicated that saliva containing high concentrations of sialic acid strongly binds influenza viruses [17], suggesting that some types of saliva contribute to virus inactivation by trapping the influenza virus. White et al. reported that submandibular-sublingual (SMSL) saliva has higher antiviral activity than parotid saliva [15]. The detailed relationship between antiviral proteins and salivary anti-IAV activity of the parotid and SMSL glands, however, is unclear.

In the present study aimed at identifying salivary proteins with anti-IAV activity, proteins in saliva collected from the parotid and SMSL glands were identified, quantified, and compared using liquid chromatography-mass spectrometry (LC-MS/MS).

## Materials and methods

### Subjects

Study participants were 12 healthy male Japanese volunteers (mean ± SD age 35.0 ± 5.0 years; range 26–46 years). Male volunteers were recruited from among the employees of the Kao Corporation in Japan; women were excluded from this study to avoid effects of the menstrual cycle on salivary flow rates [18]. Only healthy individuals without systemic disorders or metabolic diseases with good oral hygiene [19] were enrolled. The study protocol was approved by the Ethics Committee of the Kao Corporation (approval number: S201-180918), and followed the tenets of the Declaration of Helsinki. All enrollees provided written informed consent before participating in the study.

### Experimental protocol

During the experiments, which were carried out between 9:00 and 11:00 AM, participants were asked not to eat, drink, gargle, brush their teeth, or smoke for at least 1 h before, and to refrain from drinking alcohol for at least 12 h before the experiment.

With subjects in a seated position, a citric acid tablet (UHA Mikakuto Company Limited, Osaka, Japan) was placed on the tip of the tongue and salivary secretions were collected from the parotid glands and the SMSL glands using a 2-chambered suction device and collection cup, as described previously [20–23]. To avoid contamination of the SMSL saliva by parotid secretions, cotton pellets (Sekimura Limited, Tokyo, Japan) were placed on the intraoral conduit opening of the parotid glands. SMSL saliva was then collected from both sublingual and submandibular glands according to previous reports [22, 23]. The saliva from the SMSL and parotid glands was assessed on different days after centrifugation at 15,000 rpm for 15 min at 4 °C, and stored at − 80 °C until the assay was performed.

### Anti-IAV activity assays of saliva

Anti-IAV activity assays for saliva were conducted by the Research Institute for Animal Science in Biochemistry and Toxicology. Madin-Darby canine kidney cells were passaged in Eagle’s minimum essential medium (EMEM) with 5% fetal bovine serum, 0.1% sodium bicarbonate, penicillin 50 U/ml, and streptomycin 50 µg/ml. The saliva samples were preincubated for 5 min with 10,000 plaque-forming units (PFU) of human influenza virus PR8, A/Puerto Rico/8/34 (H1N1) at a 9:1 ratio. After incubation, the saliva-treated viruses were diluted by EMEM. Madin-Darby canine kidney cells seeded in 6-well plates were washed twice with phosphate-buffered saline, and then infected with the saliva-treated viruses. After 1 h absorption at 37 °C, the virus inoculum was removed, and cells were washed once with phosphate-buffered saline, and cultured for 3 days with 3 ml/well EMEM supplemented with trypsin 7.5 µg/ml, 0.1% sodium bicarbonate, and 0.75% agarose at 37 °C under 5% CO_2_. After incubation, the cells were fixed with formalin and viral plaques were visualized following staining with 0.04% methylene blue. The plaque forming rate was calculated according to the following formula: plaque forming rate (%) = PFU of saliva sample/PFU of control (phosphate-buffered saline) sample × 100. An increase in anti-IAV activity is defined as a decrease in the plaque forming rate.

### Sialic acid assay

The concentrations of total sialic acid and free sialic acid in saliva were measured using an QuantiChrom Sialic Acid Assay Kit (Bioassay Systems, Hayward, CA), using sialic acid as a standard. The assay is based on an improved Warren method [24], in which sialic acid is oxidized to formylpyruvic acid, which reacts with thiobarbituric acid to form a pink colored product. It can be quantified at λem/ex = 585/555 nm and its fluorescence is directly proportional to sialic acid concentration in the sample. The concentration of protein-bound sialic acid was calculated by subtracting the concentration of free sialic acid from the concentration of total sialic acid.

### Total protein assay

The total protein concentration of SMSL and parotid saliva was determined using the Pierce BCA Protein Assay Kit (Thermo Fisher Scientific, Waltham, MA), using BSA as a standard. Saliva samples were diluted in PBS and mixed with the BCA working reagent and the absorbance was measured at 562 nm by a microplate reader.

### Identification of saliva proteins by mass spectrometry

Salivary proteins were identified by mass spectrometry according to previously described methods [16, 25]. For ultrafiltration, 300 µL of each type of saliva was diluted with 200 µL of 100 mM Tris/HCl buffer (pH 8.5), transferred to a 3-kDa-cutoff Millipore ultrafiltration device (Merck KGaA, Darmstadt, Germany), and centrifuged at 14,000 g at 4 °C for 30 min. The retentate on the filter was then washed 3 times with 400 µL of 100 mM Tris/HCl buffer (pH 8.5). The protein concentration of the concentrated saliva was determined using the Pierce BCA Protein Assay Kit (Thermo Fisher Scientific, Waltham, MA), using BSA as a standard. Saliva samples were diluted with 100 mM Tris/HCl buffer to obtain equal amounts of protein. Saliva samples containing 100 µg protein in Tris-HCl buffer (90 µL in total) were reduced by mixing with 5 µL of 100 mM dithiothreitol at 56 °C for 45 min, and then the mixture was alkylated with 5 µL of 600 mM iodoacetamide in the dark and at room temperature for 30 min. The samples were digested with 50 µL of 0.1 µg/µL trypsin solution (Promega, Madison, WI) at 37 °C for 16 h. Finally, 10% trifluoroacetic acid (final concentration 0.3%) was added to terminate the digestion, and the sample was subjected to LC-MS/MS analysis.

Purified peptides (5 µl) were injected into a high-performance liquid chromatography system (ACQUITY UPLC: Waters, Milford, MA) connected to a triple-quadrupole mass spectrometer (TSQ Vantage; Thermo Fisher Scientific, Waltham, MA) with an ion source for electrospray ionization in multiple reaction monitoring mode. Chromatographic separation was performed on a Cadenza CD-C18 column (150 × 1 mm, 3 μm; Imtakt Corporation, Kyoto, Japan) by binary gradient elution at a 0.05 mL/min flow rate. Eluent A was 0.1% formic acid in water, and eluent B was acetonitrile containing 0.1% formic acid. Ion source (ESI) parameters were optimized as follows: spray voltage, 400 V; vaporizer temperature, 100 °C; sheath gas pressure, 40 Arb; auxiliary gas pressure, 5 Arb; and capillary temperature, 250 °C. The peak areas for each target peptide were calculated using Xcalibur Quan Browser software (Thermo Fisher Scientific, Waltham, MA). Salivary specific protein concentrations were calculated by multiplying the peak area by the total protein concentration (peak area ratio).

### Statistical analysis

Salivary anti-IAV activity, protein concentrations, and protein-bound sialic acid concentrations were compared between the SMSL and parotid saliva samples using the Wilcoxon signed-rank test. Correlations between salivary anti-IAV activity, protein-bound sialic acid concentrations, and protein concentrations were examined using the Spearman’s correlation coefficient. A *p*-value less than 0.05 was considered statistically significant. BellCurve for Excel (Social Survey Research Information, Tokyo, Japan) was used for the statistical analyses.

## Results

### Comparison of anti-IAV activities of SMSL and parotid saliva

**Fig. 1:**
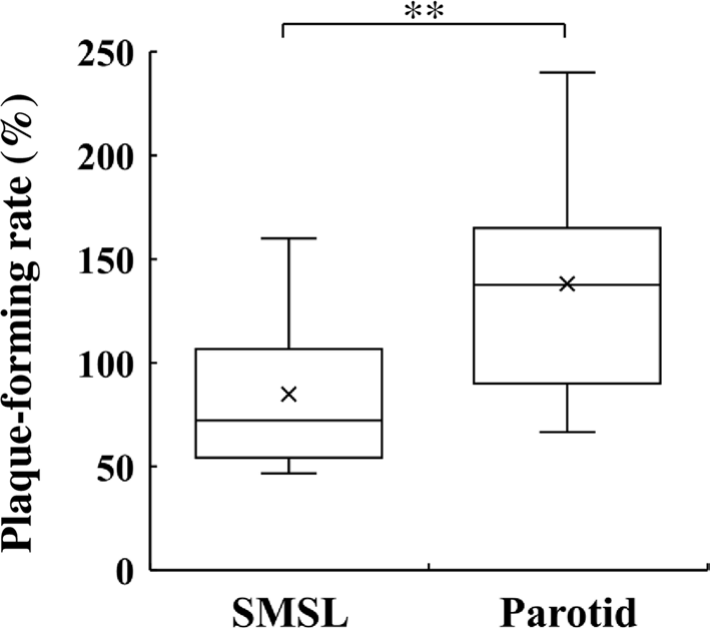
Plaque-forming rate of SMSL and parotid saliva samples collected from the orifice of the respective glands in 12 subjects. Plaque forming rate (%) = plaque forming unit (PFU) of saliva sample/PFU of control (PBS) sample × 100. Values are shown in box and whisker plots with median, interquartile, and range. Cross marks represent the mean. Wilcoxon signed-rank test; **p < 0.01

The anti-IAV virus activity of SMSL and parotid saliva was examined by plaque assay. The SMSL saliva exhibited significantly higher anti-IAV activity compared with parotid saliva, on the basis of a decrease in the plaque forming rate (Fig. 1).

### Salivary glycoproteins in SMSL and parotid saliva

**Fig. 2:**
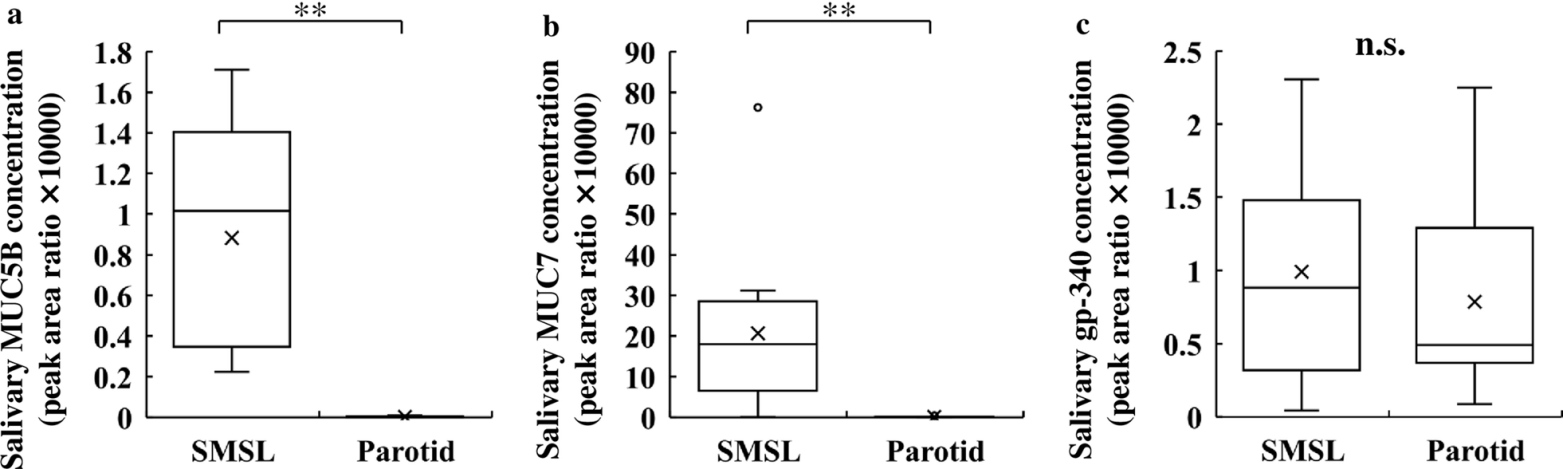
Glycoproteins, **a** mucin 5B (MUC5B), **b** mucin 7 (MUC7), and **c** glycoprotein-340 (gp-340) concentration of SMSL and parotid saliva samples collected from the orifice of the respective glands in 12 subjects. Values are shown in box and whisker plots with median, interquartile, and range. Cross mark and circle represent the means and outliers, respectively. Wilcoxon signed-rank test; **p < 0.01; n.s., not significant

Because the anti-IAV activity varied among the salivary gland types, we next examined the concentrations of salivary anti-IAV proteins by peptide analysis using LC-MS/MS. The concentrations of salivary glycoproteins such as mucin 5B, mucin 7, and gp-340 were assessed in saliva samples from each salivary gland type. Salivary mucin 5B and mucin 7 concentrations were significantly higher in the SMSL saliva than in the parotid saliva (Fig. 2a, b). The median salivary mucin 5B and mucin 7 concentrations in SMSL saliva were 1.02 and 18.06 peak area ratio, respectively, whereas those in the parotid saliva were 0.002 and 0.05 peak area ratio, respectively. The concentrations of gp-340 did not differ significantly between SMSL and parotid saliva (Fig. 2c).

### Salivary protein-bound sialic acid in SMSL and parotid saliva

**Fig. 3:**
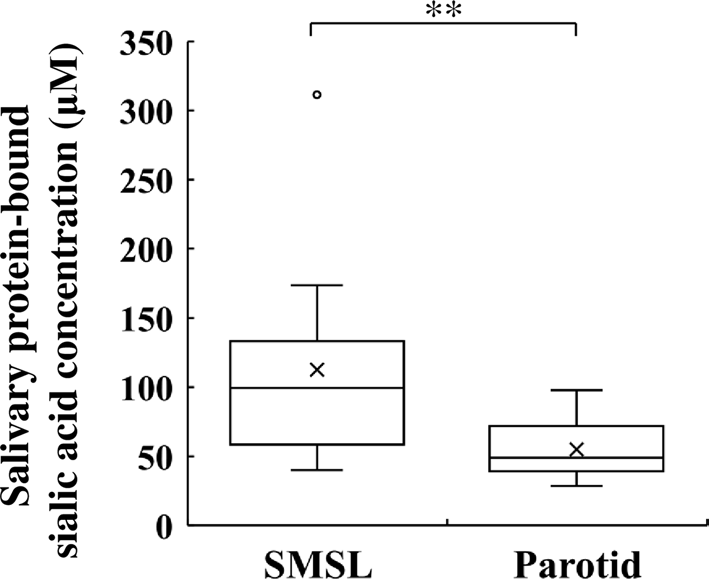
Protein-bound sialic acid concentration of SMSL and parotid saliva samples collected from the orifice of the respective glands in 12 subjects. Protein-bound sialic acid concentration = total sialic acid concentration - free sialic acid concentration. Values are shown in box and whisker plots with the median, interquartile, and range. Cross mark and circle represent the means and outliers, respectively. Wilcoxon signed-rank test; **p < 0.01

**Fig. 4:**
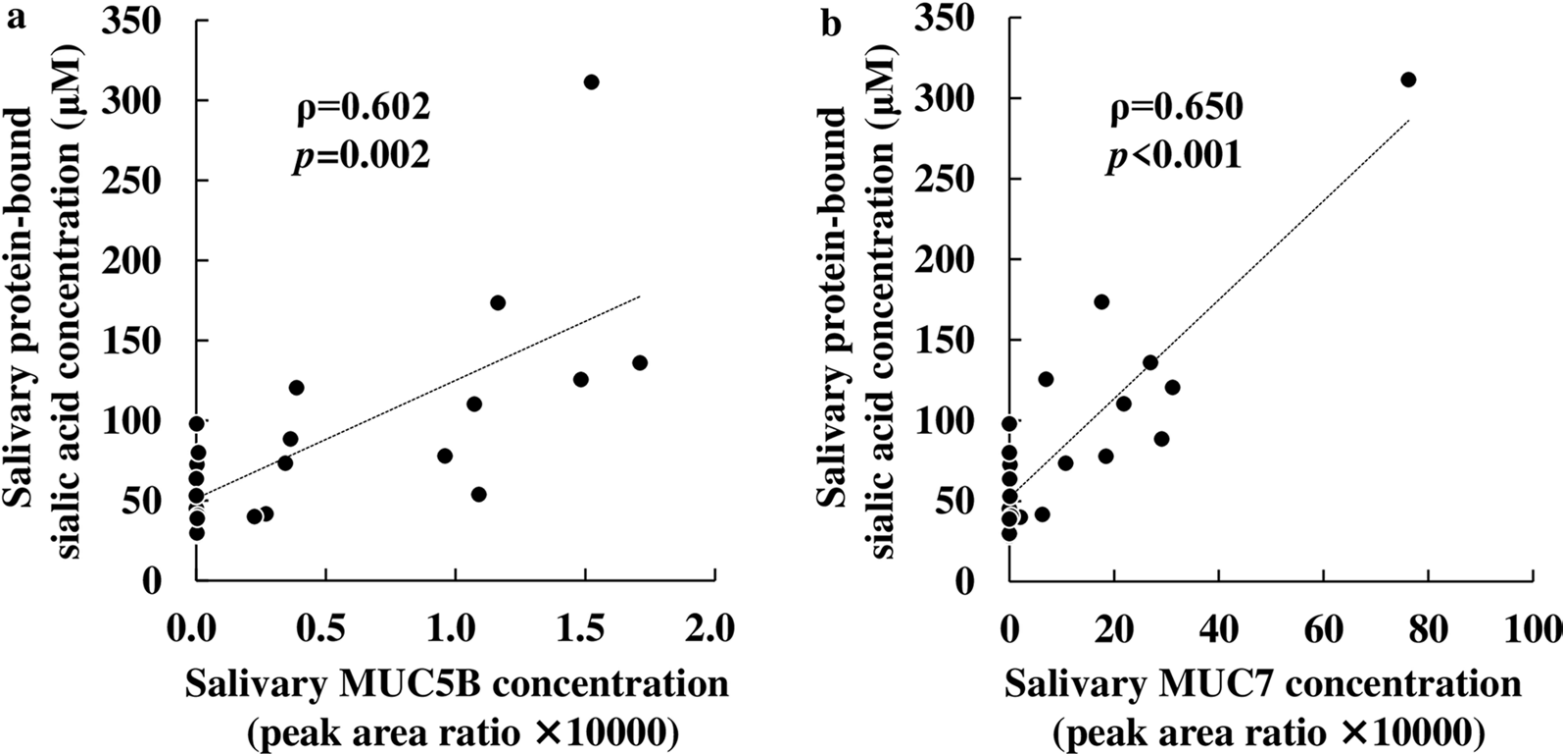
Spearman’s rank correlation coefficient (ρ) of salivary protein-bound sialic acid concentrations and salivary **a** mucin 5B (MUC5B), or **b** mucin 7 (MUC7) concentrations in 12 subjects

Because salivary anti-IAV activity and salivary mucins varied among the salivary gland types, we next examined the concentrations of protein-bound sialic acid, a component of mucin sugar chains. The concentrations of protein-bound sialic acid were assessed in saliva samples from each salivary gland type. Salivary protein-bound sialic acid concentrations were significantly higher in the SMSL saliva than in the parotid saliva (Fig. 3). The median salivary protein-bound sialic acid concentration in SMSL and parotid saliva was 99.4 and 49.0 µM, respectively. Salivary mucin 5B and mucin 7 concentrations significantly positively correlated with salivary protein-bound sialic acid (Fig. 4a, b).

### Salivary antimicrobial proteins in SMSL and parotid saliva

**Fig. 5:**
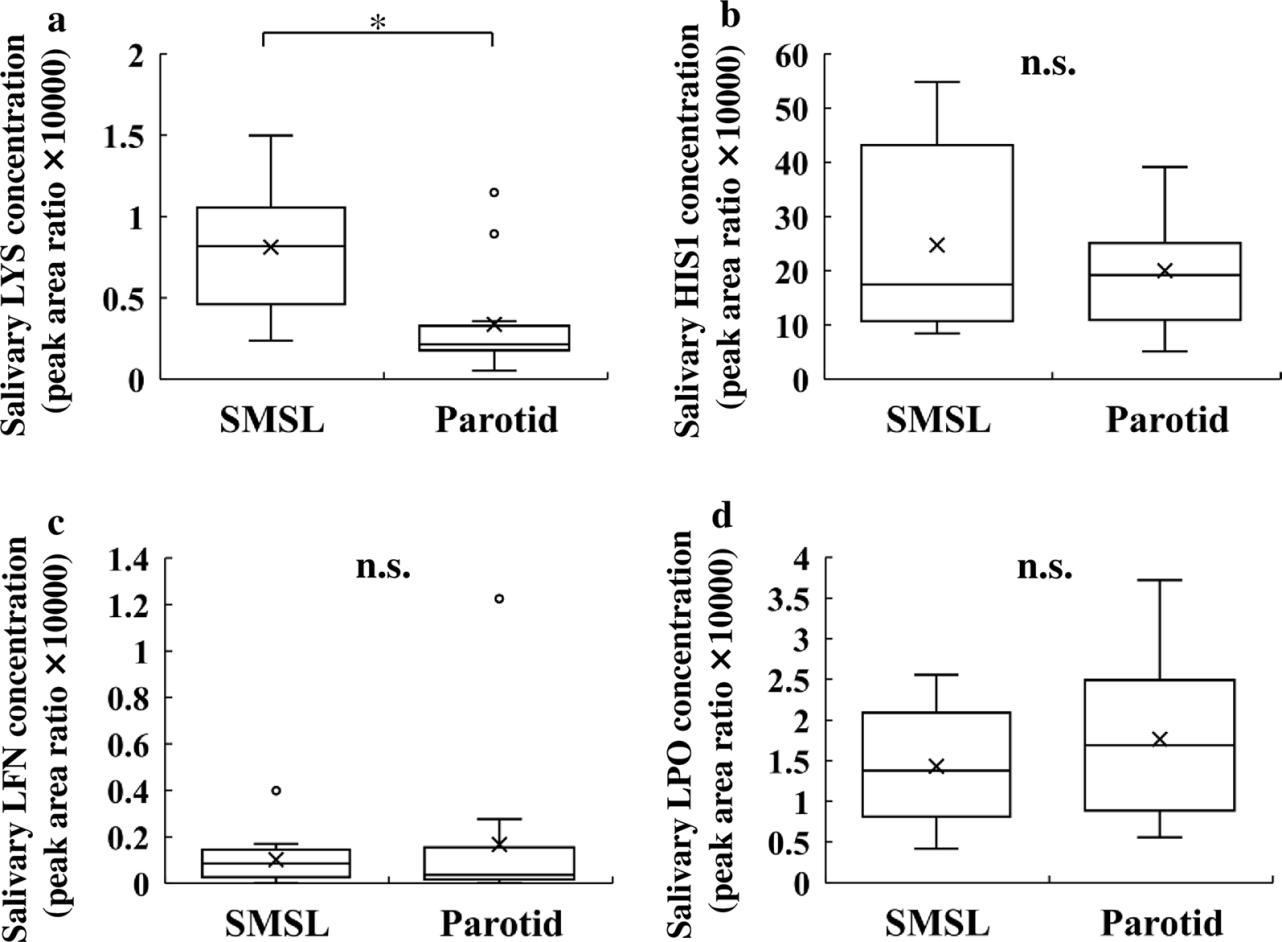
Antimicrobial proteins, **a** lysozyme C (LYS), **b** histatin-1 (HIS1), **c** lactoferrin (LFN), and **d** lactoperoxidase (LPO) concentrations in SMSL and parotid saliva samples collected from the orifice of the respective glands in 12 subjects. Values are shown in box and whisker plots with median, interquartile, and range. Cross mark and circle represent the means and outliers, respectively. Wilcoxon signed-rank test; *p < 0.05; n.s., not significant

The concentrations of salivary antimicrobial and antiviral proteins such as histain-1, lactoperoxidase, lactoferrin, and lysozyme C were assessed in saliva samples from each salivary gland type. Salivary lysozyme C concentrations were significantly higher in the SMSL saliva than in the parotid saliva (Fig. 5a). The median salivary lysozyme C concentrations in the SMSL and parotid saliva were 0.82 and 0.21 peak area ratio, respectively. The concentrations of histatin-1, lactoperoxidase, and lactoferrin did not differ significantly between SMSL and parotid saliva (Fig. 5b–d).

**Fig. 6:**
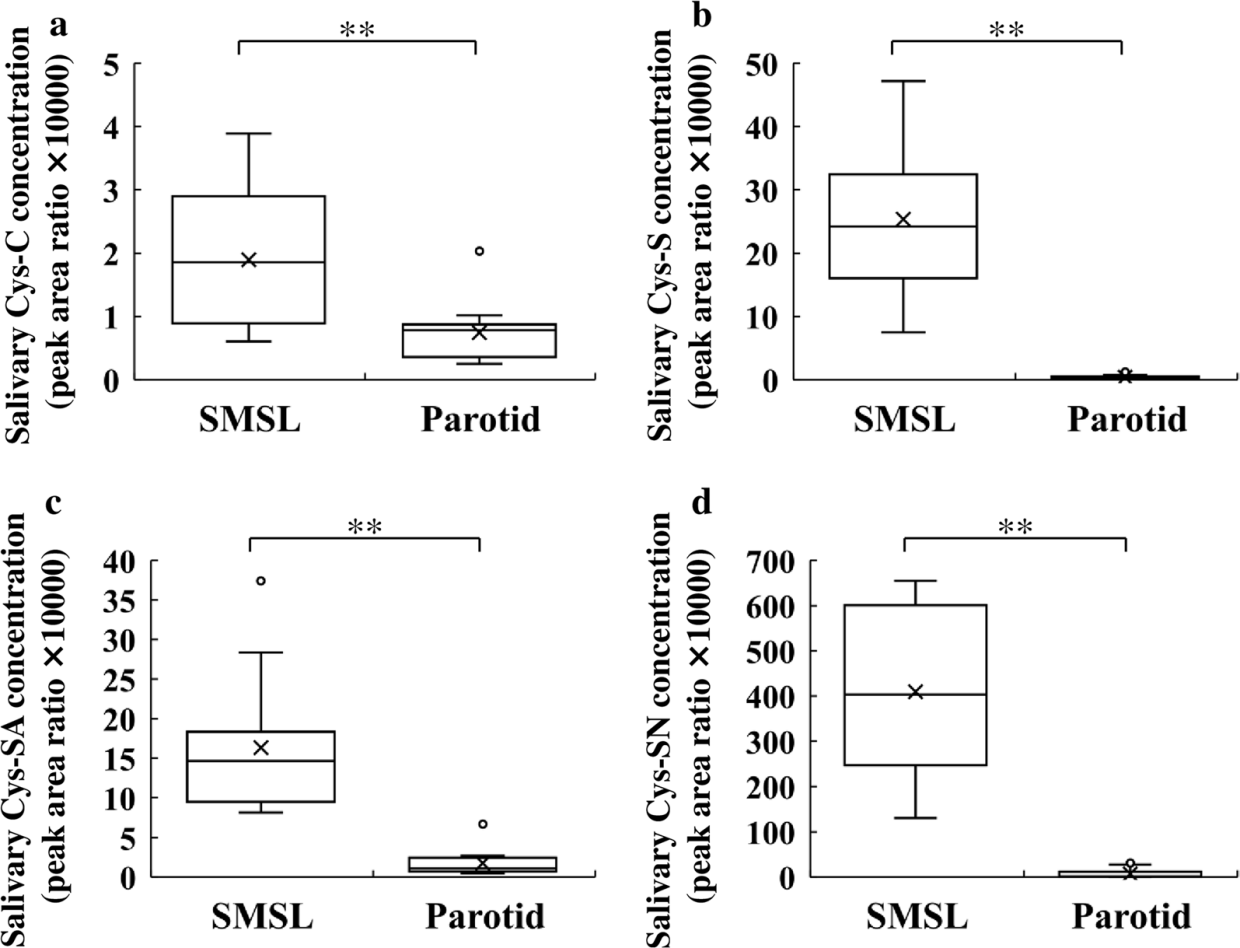
Cystatins, **a** cystatin (Cys)-C, **b** -S, **c** -SA, and **d** -SN concentrations in the SMSL and parotid saliva collected from the orifice of the respective glands in 12 subjects. Values are shown in box and whisker plots with median, interquartile, and range. Cross mark and circle represent the means and outliers, respectively. Wilcoxon signed-rank test; **p < 0.01

The concentrations of salivary cystatins were assessed in saliva samples from each salivary gland type. Salivary cystatin-C, -S, -SA, and -SN concentrations were significantly higher in SMSL saliva than in parotid saliva (Fig. 6a–d). The median salivary cystatin-C, -S, -SA, and -SN concentrations in SMSL saliva were 1.9, 24.2, 14.6, and 402.9 peak area ratio, respectively, whereas those in the parotid saliva were 0.8, 0.3, 1.1 and 1.5 peak area ratio, respectively.

### Correlation of salivary anti-IAV activity, protein-bound sialic acid, and protein concentrations

Because salivary anti-IAV and protein-bound sialic acid or protein concentrations changed inversely in each gland, we next examined the correlation of salivary anti-IAV activity, protein-bound sialic acid, and protein concentrations. The salivary plaque forming rate significantly negatively correlated with the protein-bound sialic acid, mucin 5B, mucin 7, cystatin-C, -S, and -SN concentrations (Table 1). The plaque forming rate did not significantly correlate with lysozyme C and cystatin-SA concentrations. A decrease in the plaque forming rate represents an increase in anti-IAV activity.

**Table 1:** Spearman’s rank correlation coefficient of salivary anti-IAV activity and protein-bound sialic acid and protein concentration

	PBSA	MUC5B	MUC7	LYS	Cys-C	Cys-S	Cys-SA	Cys-SN
Plaque forming rate	− 0.448*	− 0.590**	− 0.464*	− 0.249	− 0.453*	− 0.453*	− 0.380	− 0.556**

Plaque forming rate (%) = PFU of saliva sample/PFU of control (phosphate-buffered saline) sample × 100. Anti-IAV activity is defined as a decrease in the plaque forming rate

Spearman’s rank correlation coefficient; *p < 0.05; **p < 0.01

*IAV* influenza A virus; *PFU* plaque-forming units; *PBSA* protein-bound sialic acid; *MUC5B* mucin 5B; *MUC7* mucin 7; *LYS* lysozyme C; *Cys-C* cystatin C; *Cys-S* cystatin-S; *Cys-SA* cystatin-SA; *Cys-SN* cystatin-SN

## Discussion

In the present study, we simultaneously measured proteins in the saliva of the SMSL and parotid glands and their anti-IAV activity based on the IAV plaque forming rate, and analyzed their correlations. Salivary proteins from the SMSL and parotid glands with anti-IAV activity were identified. Concentrations of mucin 5B and mucin 7 were higher in SMSL saliva than in parotid saliva, which is consistent with findings from previous studies [3–5]. Mucins are highly glycosylated proteins, with a large proportion of mucin sugar chains terminating in negatively charged α-2,3 and α-2,6 N-acetylneuraminic acids, also known as sialic acids. Adhesion of viruses and bacteria is modulated by highly charged sialic acids [17]. Kobayashi et al. reported that salivary protein-bound sialic acid directly contributes to salivary anti-IAV activity [16]. We found high concentrations of protein-bound sialic acids in SMSL saliva. Protein-bound sialic acid concentrations positively correlated with salivary mucin 5B and mucin 7 concentrations. The anti-IAV activity of saliva positively correlated with the salivary protein-bound sialic acid, mucin 5B, and mucin 7 concentrations. Our findings suggest that the high anti-IAV activity of SMSL saliva is due to the effect of sialic acid-containing mucins, which are highly concentrated in SMSL saliva.

Cystatins, which are salivary cysteine protease inhibitors, have antimicrobial and protease inhibitory properties [12, 25, 26], however, anti-IAV activity of cystatins is unknown. The findings of the present study demonstrated high concentrations of cystatin family members in SMSL saliva that positively correlated with anti-IAV activity. Cystatins contain 100 amino acid residues, with no disulfide bonds or sugar chains. Therefore, cystatins may exert their anti-IAV activities through a different mechanism than sialic acid-mediated anti-IAV effects. Cystatins reportedly exhibit antiviral activity by inhibiting the proteases of the coronavirus and polio virus [13, 27]. The IAV genome does not code for the processing protease for the glycoprotein precursors involved in viral membrane fusion, IAV entry into cells is mainly determined by the trypsin-type processing proteases present in host cells that proteolytically activate the IAV fusion glycoprotein precursors. Salivary cystatins may be involved in saliva-induced anti-IAV activity by inhibiting the proteases in host cells that proteolytically activate IAV fusion glycoprotein precursors.

The SMSL saliva also contained a high concentration of lysozyme C. Lysozyme, an antimicrobial that is broadly and abundantly distributed in animal tissues and secretions, is known for its bacterial cell wall-degrading activity as well as non-enzymatic cationic features of killing bacteria [28–30]. Lysozyme exerts antiviral activity by binding to the virus and inhibiting its entry into cells via its cationic nature [29]. The effect of lysozyme on IAV is unknown, but salivary lysozyme may be involved in the anti-IAV activity by binding to IAV and inhibiting IAV entry into the host cells. Because the correlation coefficients of salivary anti-IAV activity and lysozyme concentration were low, the contribution of lysozyme to salivary anti-IAV activity is likely small.

Human saliva contains high concentrations of both antibacterial and antiviral proteins, including lactoferrin, lysozyme, mucins, gp-340, and serum leukocyte protein inhibitor [14]. The protein content differs in parotid and SMSL saliva [3–5], suggesting that the functions of the SMSL and parotid saliva also differ. In the present study, SMSL saliva exhibited higher antiviral activity than the parotid saliva, and thus promotion of SMSL saliva secretion may be effective toward preventing influenza infection. Carbonic acid is useful for stimulating SMSL salivary secretion [31], and may thus be useful in the prevention of influenza infection. Because the salivary protein cystatin has inhibitory effects on coronavirus [27], promoting SMSL saliva secretion may also be effective against COVID-19, but further research is needed.

## Conclusion

In conclusion, human SMSL saliva had higher anti-IAV activity than parotid saliva, the SMSL saliva had higher concentrations of mucin 5B, mucin 7, and cystatins, and these proteins positively correlated with the anti-IAV activity of the saliva. These observations suggest that promotion of SMSL saliva secretion may be effective toward preventing influenza infection.

## Data Availability

The datasets obtained and analyzed during the current study are available from the corresponding author on reasonable request.

## Abbreviations

IAV: Influenza A virus
LC-MS/MS: Liquid chromatography-mass spectrometry
PFU: Plaque-forming units
SMSL: Submandibular-sublingual
